# Nonequilibrium Energy Transfer at Nanoscale: A Unified Theory from Weak to Strong Coupling

**DOI:** 10.1038/srep11787

**Published:** 2015-07-08

**Authors:** Chen Wang, Jie Ren, Jianshu Cao

**Affiliations:** 1 Department of Chemistry, Massachusetts Institute of Technology, 77 Massachusetts Avenue, Cambridge, MA 02139, USA; 2 Singapore-MIT Alliance for Research and Technology, 1 CREATE Way, Singapore 138602, Singapore; 3 Department of Physics, Hangzhou Dianzi University, Hangzhou, Zhejiang 310018, China; 4 Center for Phononics and Thermal Energy Science, School of Physics Science and Engineering, Tongji University, 200092 Shanghai, P. R. China

## Abstract

Unraveling the microscopic mechanism of quantum energy transfer across two-level systems provides crucial insights to the optimal design and potential applications of low-dimensional nanodevices. Here, we study the non-equilibrium spin-boson model as a minimal prototype and develop a fluctuation-decoupled quantum master equation approach that is valid ranging from the weak to the strong system-bath coupling regime. The exact expression of energy flux is analytically established, which dissects the energy transfer as multiple boson processes with even and odd parity. Our analysis provides a unified interpretation of several observations, including coherence-enhanced heat flux and negative differential thermal conductance. The results will have broad implications for the fine control of energy transfer in nano-structural devices.

Energy dissipation has become a severe bottleneck to the sustainability of any modern economy^1^. To address this issue, efficient energy transfer and the corresponding smart control and detection at nanoscale have created unprecedented opportunities and challenges^2^,^3^,^4^,^5^. Therefore, understanding and controlling energy transfer in low-dimensional systems is of significant importance not only in fundamental researches but also in practical applications^6^,^7^,^8^,^9^. For typical energy transport far from equilibrium, two baths should be included with thermodynamic bias (e.g. temperature bias), as shown in Fig. 1. The prototype paradigm is termed as the nonequilibrium spin boson model, given by


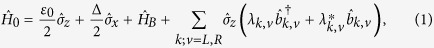


where the two-level system (TLS) is represented by Pauli matrices 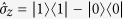 and 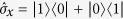, with *ε*_0_ the energy spacing and Δ the tunneling strength between the TLS. 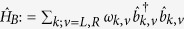 denotes the bosonic baths with 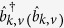 creating (annihilating) one boson with energy *ω*_*k,v*_ and momentum *k* in the *v*th bath. The last term describes the spin-boson interaction with *λ*_*k,v*_ the coupling strength. In the long time limit, the system reaches the nonequilibrium steady state (with stable energy flow).

For NESB, the TLS can manifest itself as impurity magnets, anharmonic molecules, excitons, cold atoms, low-energy band structures, *etc*. Bosonic baths can register as electromagnetic environments, lattice vibrations, Luttinger liquid, magnons, *etc*. Hence, the NESB has already found widespread applications in fertile frontiers. Particularly, In phononics^5^, NESB describes the phononic energy transfer in anharmonic molecular junctions^10^,^11^,^12^,^13^,^14^,^15^,^16^,^17^, and can be regarded as a special realization of the famous Caldeira-Leggett model^18^. In many-body physics, NESB describes the novel Kondo physics and nonequilibrium phase transitions^19^,^20^. In spin caloritronics, NESB describes the nontrivial spin Seebeck effects that pave the way for thermal-driven spin diode and transistor^21^. In quantum biology, NESB models the exciton transfer embedded in the photosynthetic complexes^7^,^9^,^22^,^23^,^24^. Also, NESB describes electromagnetic transport through superconducting circuits^25^ and photonic waveguides with a local impurity^26^. Moreover, this generic model can be extended to one dimensional spin chains at ultra-low temperatures^27^.

Theoretically, many approaches have been proposed to explore energy transfer in NESB, but each approach works with limitations. Typically, in the weak spin-boson coupling regime, the Redfield equation applies and gives the resonant energy transfer and additive contributions of separate baths^12^,^16^. While in the strong spin-boson coupling regime, the nonequilibrium version of the noninteracting-blip approximation (NIBA) equation applies and provides the off-resonant steady energy transfer and non-additive picture^14^,^28^,^29^, which is usually based on the Born approximation in the polaron framework. Note the traditional NIBA of a single bath spin-boson model is consistent with the Redfield scheme in the weak-coupling regime^30^. This is distinct from the nonequilibrium NIBA, which only applies in the strong-coupling limit^29^. Moreover, for the negative differential thermal conductance (NDTC), the nonequilibrium NIBA scheme claims its appearance in the strong coupling for NESB, whereas the Redfield scheme predicts its absence in the weak coupling^14^. Similar limitations between these two schemes also occur in the high order flux-fluctuations as well as in the geometric-phase-induced energy transfer^29^. Although some numerical simulations, e.g. path-integral monte carlo and multi-configuration time-dependent Hartree (MCTDH) method^20^,^31^, have recently been carried out, which attempt to exactly calculate the energy transfer in NESB, they all have their practical limitations or require expensive computations. Moreover, numerical approaches may not provide clear physical insights to the underlying energy transfer mechanism.

To solve the long-standing challenge and answer these important questions, we present a nonequilibrium polaron-transformed Redfield equation (NE-PTRE), which is based on the fluctuation-decoupling method perturbing the spin-boson interaction in the polaron framework. This approach is capable of bridging the energy transfer pictures of NESB from weak to strong coupling regimes. Then, the energy transfer in NESB is clearly unraveled as multi-boson processes, which are classified by the odd-even parity, with the sequential- and co-tunneling behaviors as two lowest order contributions. To exemplify the power of our unified theory, we derive the analytical expression of energy flux that dissects the transfer processes systematically, and show that this unified flux expression reduces to the NIBA at strong coupling limit and to the Redfield one at the weak coupling limit, respectively. Moreover, we investigate NDTC and identify its absence over wide range of the temperature bias, even in the intermediate and strong coupling regimes, which corrects the previous observation of NDTC under the NIBA in the classical limit^14^.

## Results

### Fluctuation-decoupling based quantum master equation

Based on the canonical transformation 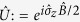 to the NESB Hamiltonian at Eq. (1), a new transformed Hamiltonian is obtained as 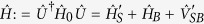, with the new system Hamiltonian


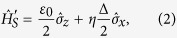


and the new system-bath interaction


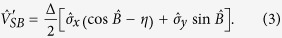



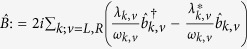
 is the collective momentum operator of bosonic baths, and the renormalization factor 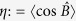 is specified as


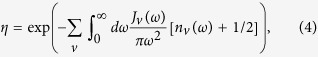


where 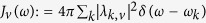 is the 1th bath spectrum and *n*_*v*_(*ω*): = 1/[exp(*β*_*v*_*ω*_*v*_) − 1] denotes the corresponding Bose-Einstein distribution with *β*_*v*_ = 1/*k*_*b*_*T*_*v*_ the inverse temperature. Clearly, the renormalization factor *η* vanishes to 0 for the strong system-bath coupling strength but approaches to 1 at the weak coupling limit.

Traditionally, many methods, including the NIBA^28^,^29^, directly treat the interaction 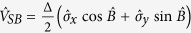 as a perturbation. However, we note that generally 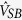 can not behave as a perturbation due to the non-negligible expectation 
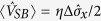
, expect for weak inter-site tunneling (Δ → 0) or strong system-bath coupling (*η* → 0). Nevertheless, the fluctuation around the expectation value 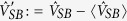 may be safely treated by the second order perturbation, regardless of the tunneling and coupling strength. Therefore, by means of this fluctuation-decoupling scheme, the new system-bath interaction may be reliably perturbed regardless of the coupling strength. It should be acknowledged that similar schemes with the spirit of “fluctuation decoupling” were also carried out in other excellent works^32^,^33^,^34^,^35^,^36^,^37^. The contribution from second order terms of 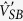 is found to be small compared to 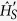. Moreover, from the quantum dynamics, the second order perturbation of 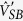 was excellently applicable to capture dynamical behaviors, particularly in the long time limit^32^,^33^. We would also like to point out that although the polaron transformation is adopted conventionally as well as in our present study, the fluctuation-decoupling scheme is not limited to this transformation but refers to general scheme that subtracts the expectation of the system-bath coupling from itself and compensates the expectation back to the system’s Hamiltonian so that the new system-bath interaction may be reliably perturbed.

In energy transfer studies, the spectrum can be usually considered as 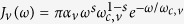 with *α*_*v*_ 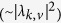 the coupling strength and *ω*_*c,v*_ he cutoff frequency. Without loss of generality, we choose the typical super-Ohmic spectrum *s* = 3 for consideration in this paper^2^. Then, the renormalization factor at Eq. (4) is specified as 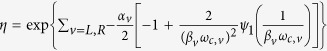, where the special function 
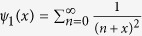
 is the trigamma function. It should be noted that if we select the bosonic baths as the Ohmic case *s* = 1, the renormalization factor expressed at Eq. (4) will always approach to zero regardless of the system-bath coupling strength, and the expectation of the system-bath interaction at Eq. (3) 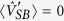. As such, the NE-PTRE based on the fluctuation-decoupling scheme, will be equivalent to the nonequilibrium NIBA^28^,^29^.

Fluctuation-decoupling is the key step, based on which we are able to apply various perturbative methods to proceed. Here, we adopt the nonequilibrium polaron-transformed Redfield equation and obtain (see the Method):


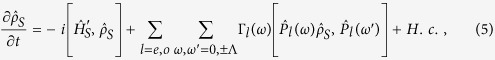


where 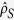 is the reduced density matrix for the TLS in the polaron framework, 
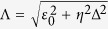
 is the energy gap of the renormalized TLS in its eigenspace, and 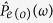 is the measuring projector in the eigen-basis obtained from the evolution of spin matrices 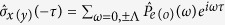. The subscript *e*(*o*) denotes the even (odd) parity of transfer dynamics. Γ_*l*_(*ω*) with *l* = *e*, *o* has the meaning of transition rate that we will discuss later in detail.

### Parity classified transfer processes

As the crucial observation, the transition rates are expressed as 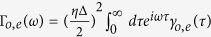 (see the Method), where the correlation functions are specified by


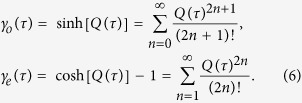


The boson propagator 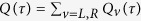 with 
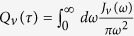
 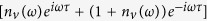 describes the bosonic absorptions and emissions that constitute the energy transfer. Clearly, the multi-boson processes are classified by the odd and even propagators, with each order fully captured by the corresponding Taylor expansion systematically.

**Figure 2:**
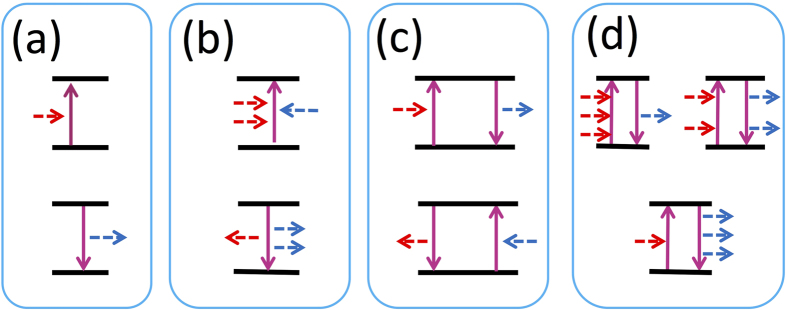
Representative processes in multi-boson assisted energy transfer: (**a**) single boson involved sequential process; (**b**) three-boson involved “tri-tunneling” process; (**c**) two-boson “cotunneling” process; (**d**) four-boson involved collective process.

Specifically, *γ*_o_(*τ*) describes the processes involving odd boson numbers. The lowest order contribution is the sequential-tunneling [see Fig. 2(a)], expressed by 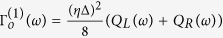
12,14,16, with 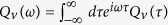 and 
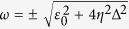
. This means that the relaxation and excitation of the TLS is influenced by the *L* and *R* baths separately, i.e., additively. Further, the higher order, called as “tri-tunneling” [Fig. 2(b)], is exhibited as 
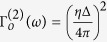
 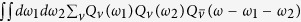, with 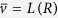 for *v* = *R*(*L*), where the baths act non-additively and off-resonantly. This highly non-trivial term explicitly demonstrates the collective transfer process with different contributions from two baths.

Correspondingly, *γ*_*e*_(*τ*) describes processes of even boson number participating in the energy transfer processes. The lowest order includes the co-tunneling effect^38^ [see Fig. 2(c)]. It contributes to the transition rate as 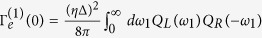. This implies that when the left bath releases energy *ω*_1_, the right bath absorbs the same quanta simultaneously, leaving the TLS unchanged. Clearly, two baths are involved non-additively. The corresponding higher order term can also be obtained systematically [see Fig. 2(d)]. As a result, we can dissect the contribution of each order of boson excitations to the energy transfer based on the expansions, and the underlying multi-boson transfer mechanism can be systematically exploited.

### Unified energy flux from weak to strong couplings

To exploit the dynamical processes corresponding to the correlation functions in Eq. (6), we introduce the rate 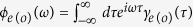 in the frequency domain. As such, when rewriting 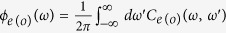, we are able to extract the corresponding kernel functions


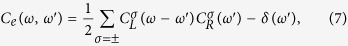



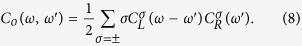


where 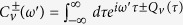 describes the rate density of the *v*th bath absorbing (emitting) energy *ω* (−*ω*), obeying the detailed balance relation as 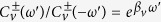.

These kernel functions provide the other way of understanding the odd-even parity assisted energy tunneling processes that incorporate two baths non-additively. Physically, *C*_*e*(*o*)_(*ω, ω*′) describes that when the TLS releases energy * ω* by relaxing from the excited state to the ground one, the right bath absorbs energy *ω*′ and the left one obtains the left *ω* − *ω*′ if *ω* *>* *ω*′ or supply the compensation if *ω* *<* *ω*′. And *C*_*e*(*o*)_(−*ω, ω*′) describes similar dynamical processes for the TLS jumping from the ground state to the exciting one. While *ϕ*_*e*(*o*)_(*ω*) is the summation behavior of these corresponding microscopic processes.

In many energy transfer studies, the resonant case (*ε* = 0) is of prime interest. The steady state populations can be obtained in the local basis as *P*_11_ = *P*_00_ = 1/2, and the coherence is


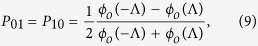


with the energy gap Λ = *η*Δ and 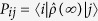. Combined with the the counting field^16^,^29^,^39^, we obtain the energy flux as


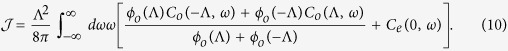


It is interesting to find that in the odd parity subspace, as the TLS relaxes energy Λ, the baths show collective contribution *C*_*o*_(−Λ*, ω*) to the flux with the weight 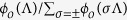. Similarly, when the TLS is excited by an energy Λ, *C*_*o*_(Λ*, ω*) is contributed to the flux with the corresponding subspace weight as 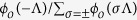. While for the even parity subspace, the TLS energy is unchanged, with the contribution *C*_*e*_(0*, ω*) to the flux. This unified energy flux expression clearly uncovers that two parity-classified sub-processes both contribute to the energy transfer, whereas the Redfield approach merely includes the lowest odd order and the NIBA only considers the even order. More details are analytically discussed in the following:

In the weak coupling limit, one only needs to keep the leading order of the correlation function as *O*(*α*_*v*_) so that the renormalization factor is simplified to *η* ≈ 1 and Λ = Δ. Hence, the kernel function with even parity *C*_*e*_ (0, *ω*) = 0 and the odd one becomes 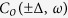 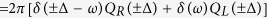. The unified energy flux reduces to the resonant energy transfer


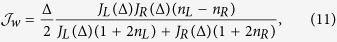


with *n*_*v*_ = *n*_*v*_(Δ), which is consistent with previous results of Redfield approach^12^,^16^. While in the strong coupling limit, multiple bosons are excited from baths, and both the renormalization factor *η* and the eigen-energy gap of the TLS Λ become zero. Hence, two subspace kernel functions at Eq. (7) show equal weight. The energy flux can be finally expressed as


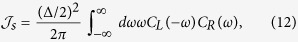


with the probability density of the *v*th bath


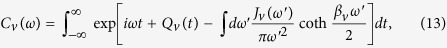


which correctly recovers the nonequilibrium NIBA result^14^,^28^. It should be noted that in the strong interaction regime, *η* → 0, and we have 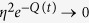 that is independent of time *t*. However, *η*^2^*e*^*Q*(*t*)^ keeps finite positive. This directly results in the vanishing transition rate Γ_0_(0) = 0 and a finite transition rate 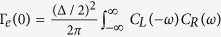, which means that only even number of phonon scattering processes contributed to the energy transfer. This clearly shows that nonequilibrium NIBA method contains the even parity of the energy transfer process but misses the odd order.

**Figure 3:**
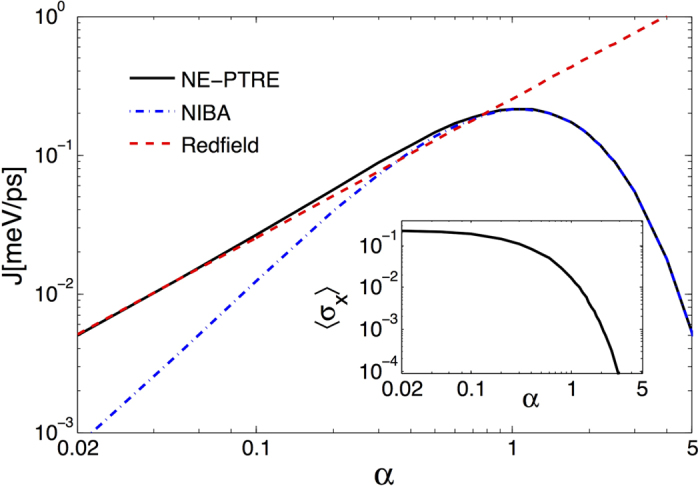
The energy flux and quantum coherence represented by 〈*σ*_*x*_〉, as functions of the coupling strength. The solid black line is from the NE-PTRE, which unifies the Redfield result at the weak coupling (the red dashed line) and the NIBA result at the strong coupling (the dot-dashed blue line). The deviation of the unified energy flux from the NIBA result at small *α* is characterized by the quantum coherence 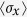 (inset). Parameters are given by *ε*_0_ = 0, ******Δ = 5.22 meV, *ω*_*c*_ = 26.1 meV, *T*_*L*_ = 150 K and *T*_*R*_ = 90 K.

Next, we plot the energy flux of Eq. (10) in Fig. 3, which first shows linear increase with the system-bath coupling at weak regime, consistent with the Redfield. After reaching a maximum, the energy flux decreases monotonically in the strong coupling regime, of which the profile coincide with the NIBA. The discrepancy of the NIBA and our NE-PTRE is due to the improper ignorance of quantum coherence 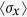 of the TLS in NIBA (see also Eq. (2), in which the term containing *σ*_*x*_ is absent in the NIBA method). This coherence term describes the effective tunneling within TLS so that it enhances the energy transfer compared to the NIBA that ignores it.

Therefore, we conclude that the unified energy flux expression of Eq. (10) provides a comprehensive interpretation for energy transfer in NESB, because the fluctuation-decoupling scheme not only describes the coherent system-bath coupling from weak to strong regimes, but also correctly captures the coherence within the TLS.

### Absence of negative differential thermal conductance

NDTC, a typical feature in energy transport, has been extensively studied in phononic devices^5^. In particular, NDTC has also been exploited in molecular junctions, represented by the NESB. By adopting nonequilibrium NIBA in the Marcus limit, i.e. high temperature baths, it was reported that NDTC is absent in the weak coupling but emerges in the strong coupling regime^14^. However, what happens at the intermediate coupling regime is unclear. Moreover, it is questionable that whether NDTC is still presented in the comparatively low temperature regime.

Marcus theory was originally proposed to study semi-classical electron transfer rates in the donor-acceptor species^2^,^40^. In previous works of energy transport^17^,^28^,^29^, the system dynamics with the Marcus limit is described by the rate equation based on the nonequilibrium NIBA, i.e. Eq. (3) in Ref. 29. In the high temperature limit, it is known that *n*_*v*_(*ω*) ≈ 1/(*β*_*v*_*ω*) and the low frequency domain of bosonic baths dominates the evolution, which corresponds to the short-time expansion 1 − cos*ωτ* ≈ *ω*^2^*τ*^2^/2 and sin*ωτ* ≈ *ωτ*^12^,^14^,^29^. Thus the Gaussian decay of the the probability density is given by 
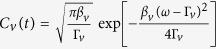
, which is as the same as derived from Eq. (13) even under biased condition. The renormalized coupling strength is 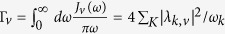. Hence, the energy flux can be obtained accordingly with the help of counting field^29^. However, this limiting picture may be modified if the temperatures of bosonic baths become low, when the quantum effect will be included to make the probability density non-Gaussian.

**Figure 4:**
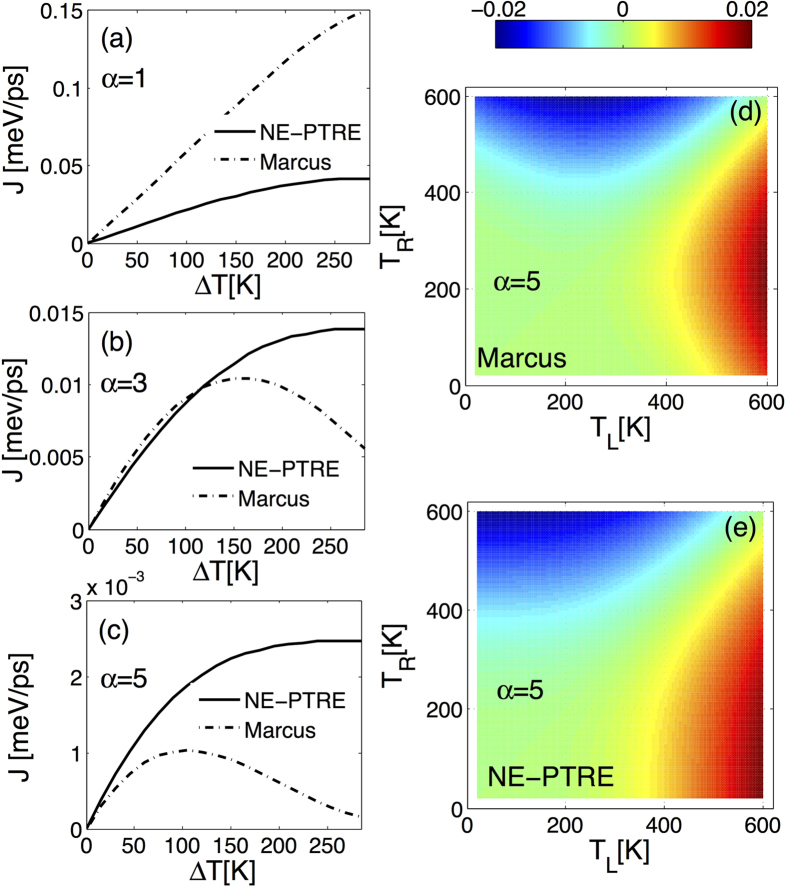
Energy flux in the intermediate and strong system-bath coupling regimes by tuning the right bath temperature in (**a**–**c**), and the birdeye view of the energy flux by varying the two bath temperatures in (**d**–**e**). The parameters are given by Δ = 10 meV, *ε*_0_ = 10 meV, *ω*_*c*_ = 26.1 meV, *T*_*L*_ = 300 K and *T*_*R*_ = *T*_*L*_ − Δ*T*.

Therefore, we re-examine the NDTC by the NE-PTRE in Fig. 4(a–c). In the intermediate coupling regime (*α* = 1), the energy flux increases monotonically by enlarging the temperature bias (Δ*T* = *T*_*L*_ − *T*_*R*_), both for the NE-PTRE and the NIBA of the classical (Marcus) limit. As the coupling is strengthened further into the strong coupling regime (i.e. *α* = 3 and *α* = 5), NDTC was found to be apparent upon the Marcus limit^14^. However, no turnover signal is found based on the NE-PTRE. In the strong coupling limit, *η* → 0 so that our method reduces to the NIBA, thus the discrepancy comes from the Marcus approximation. It should be noted that from Fig. 4(b,c), the qualitative deviation occurs at the large temperature bias. This means that the temperature of the right bath is rather low, and quantum effect as such low temperatures may change behaviors of the correlation functions. To further clarify the absence of NDTC at the deep strong coupling regime, the birdeye view contours of energy flux are compared with and without Marcus limit [see Fig. 4(d,e)]. It is shown that the turnover behavior appears within the Marcus framework, by tuning either *T*_*L*_ or *T*_*R*_ (see Fig. 4(d)), whereas it never emerges with rigorous calculations [see Fig. 4(e)]. In fact, this result clearly demonstrates that the NDTC in the Marcus limit occurs at large temperature bias Δ*T* = *T*_*L*_ − *T*_*R*_ with either *T*_*R*_ or *T*_*L*_ at very low temperature, where the high temperature precondition of the Marcus framework may break down. Thus the NDTC observed in the NIBA scheme with Marcus assumption is merely an artifact.

Hence, we conclude that by tuning one bath temperature, NDTC is absent across a wide range of the temperature bias in the NESB model even in the strong system-bath coupling limit. Finally, we would like to note if we allow to change two temperatures simultaneously, NDTC can still occur in NESB. Also, NDTC is not exclusive to the strong coupling limit generally, but can even exist in the weak coupling regime if the system is hybridized with fermion-spin-boson couplings^21^,^41^.

## Discussion

Steady state energy transfer in nonequilibrium spin boson systems has been studied both theoretically and numerically by various approaches (e.g. Redfield, noneuqilibrium NIBA, MCTDH). However, until the present work there is no existing analytically unified theory to explicitly unravel the underlying physics, especially for the expression of energy flux, which may be directly measured by practical experiments. In particular, the novel role of parity to the detailed transfer process has not been exposed before.

Hence, we apply the nonequilibrium polaron-transformed Redfield equation based on fluctuation decoupling to exploit the unified energy transfer theory. It should be noted that polaron-transformation-based quantum master equation has been previously proposed by R. J. Silbey *et al.*^34^,^35^ in the equilibrium spin-boson model with only a single bath under the Born-Markov approximation. Then, it was extended to study the non-Markovian dynamics^32^ and include the correlated bath effect^36^. Recently, it was confirmed both from the equilibrium statistics and quantum dynamics that the Markovian master equation combined with the polaron transformation can be accurately utilized in the fast bath regime^33^,^37^. However, the nonequilibrium energy transfer with thermodynamic bias has never been touched within polaron framework, mainly lack of flux counting tool (e.g. full counting statistics). In this paper we focus on the nonequilibrium transport in fast bath regime. The Markovian master equation is believed to be applicable, and will be proved in future numerical exact work.

In conclusion, we have unified the energy transfer mechanisms in the nonequilibrium spin-boson model from weak to strong coupling regimes. Specifically, we have characterized energy transfer as multi-boson processes that are classified by the odd-even parity. We have analytically obtained the energy flux expression in Eq. (10), which explicitly unifies the analytic results from the weak-coupling Redfield scheme and the strong-coupling NIBA scheme. Moreover, enhancement of the energy flux at the intermediate coupling regime has been identified, which results from the persistence of coherent tunneling within the TLS but is unexpectedly ignored in the nonequilibrium NIBA. Other relevant controversial problems of energy transfer in NESB have also been systematically resolved. We believe our results provide a comprehensive interpretation of previous works and can have broad implications for smart control of energy and information in low-dimensional nanodevices.

## Methods

### Nonequilibrium polaron-transformed Redfield equation

The model of a TLS interacting with two separate baths after polaron transformation is described by


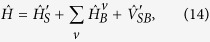


where 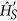 denotes the system Hamiltonian at Eq. (2), 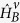 models the *v* bath, and 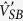 is the interaction between the system and the bosonic baths, as shown at Eq. (3). Assuming the strength of the system-bath coupling is weak compared to the intrinsic energy scale of the system, people usually apply Born-Markov approximation to derive the second-order master equation^2^


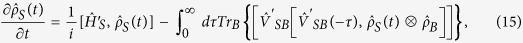


where 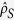 is the reduced system’s density operator,
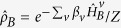
 is the canonical distribution of the baths, and 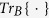 traces off the degree of freedom of baths. As the bias is applied on two baths, the quantum system is driven from the equilibrium state to the nonequilibrium steady state, which spontaneously generates the energy or particle flux.

By tracing the degrees of freedom of bosonic baths, the nonequilibrium polaron-transformed Redfield equation at Eq. (5) can be fully recovered. *P*_*l*_(*ω*) is the eigen-state projector from the evolution of the Pauli operators as 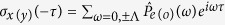 with the energy gap 
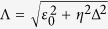
, the eigen-states 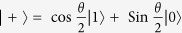,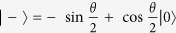 and 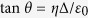 In the even parity, they are specified as 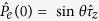, 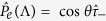, and 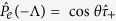, with 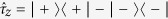 and 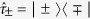. Similarly in the odd parity, they become 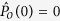, 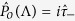 and 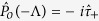.

Considering the expression of 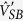 at Eq. (3) and the structures of system-bath interaction terms (for example, one of the four is 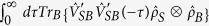), we can calculate out the transition rates Γ_*o*,*e*_ mediated by the TLS and readily obtain the correlation function


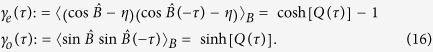


The transition rates are expressed by the Fourier transform of the correlation functions: 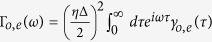, where the bath collective momentum operator is 
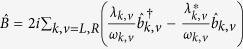
 and the boson propagator is 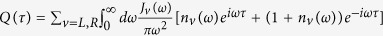.

### Definition of the steady state energy flux

In the Liouville space, the quantum master equation combined with the full counting statistics can be expressed as (see, e.g.,^39^,^42^,^43^,^44^)


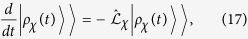


with 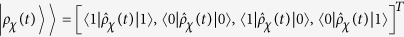 in the vector form, and 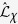 the super-operator. The generating function is obtained by


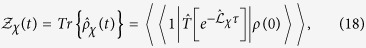


where 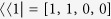, 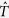 is the time-ordering operator and 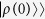 is the vector of density matrix of the initial system. Energy transfer behaviors in the long time limit are of our prime interest in the present paper. They are controlled by the ground state of 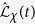, with the ground state energy as *E*_0_(*χ*) having the smallest real part. Hence, the generating function is simplified to 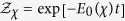. Then the steady state cumulant generating function can be derived by 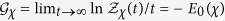, which finally generates the steady energy flux as 
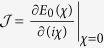


Alternatively, based on the Eq. (17) the steady state solution can be expressed as 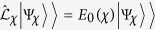, with 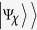 the corresponding right ground state. Taking the derivative of *iχ* at two sides results in


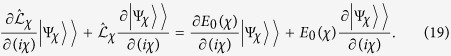


When *χ* = 0, it is known that *E*_0_ = 0, 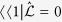 and 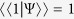, where 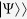 is equal to the vector of the density matrix at steady state. As a result, 
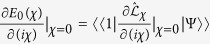
 and the energy flux is re-expressed as


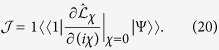


## Additional Information

**How to cite this article**: Wang, C. *et al.* Nonequilibrium Energy Transfer at Nanoscale: A Unified Theory from Weak to Strong Coupling. *Sci. Rep.* **5**, 11787; doi: 10.1038/srep11787 (2015).

## Figures and Tables

**Figure 1:**
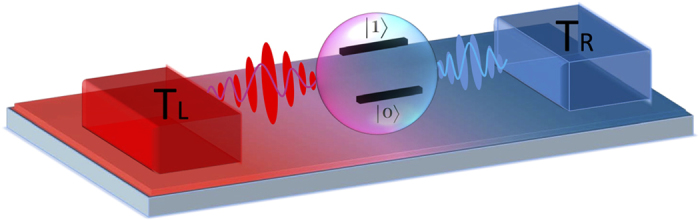
Schematic illustration of the nonequilibrium spin-boson model composed by central two-level nanodevice connecting to two separate bosonic baths with temperature *T*_*L*_ and *T*_*R*_ respectively.
